# Healthcare providers’ readiness for electronic health record adoption: a cross-sectional study during pre-implementation phase

**DOI:** 10.1186/s12913-022-07688-x

**Published:** 2022-03-02

**Authors:** Habtamu Setegn Ngusie, Sisay Yitayih Kassie, Alex Ayenew Chereka, Ermias Bekele Enyew

**Affiliations:** Department of Health Informatics, College of Health Sciences, Mettu University, P.O.Box:318, Mettu, Ethiopia

**Keywords:** E-health, Electronic health record, Electronic medical record, EHR adoption, Healthcare provider, Healthcare providers readiness, Health information technology, Pre-implementation phase, Southwest Ethiopia

## Abstract

**Background:**

The adoption of an electronic health record (EHR) in the healthcare system has the potential to make healthcare service delivery effective and efficient by providing accurate, up-to-date, and complete information. Despite its great importance, the adoptions of EHR in low-income country settings, like Ethiopia, were lagging and increasingly failed. Assessing the readiness of stakeholders before the actual adoption of EHR is considered the prominent solution to tackle the problem. However, little is known about healthcare providers’ EHR readiness in this study setting. Accordingly, this research was conducted aiming at examining healthcare providers’ readiness for EHR adoption and associated factors in southwestern Ethiopia.

**Methods:**

An institutional-based cross-sectional study was conducted from September 1 to October 30, 2021. A total of 423 healthcare providers working in public hospitals were selected using a simple random sampling technique. Multivariable logistic regression was fitted to identify determinant factors of overall healthcare providers’ readiness after the other covariates were controlled.

**Result:**

In this study, the overall good readiness level of EHR adoption was 52.8% (*n* = 204) [95% CI of 47.9% to 56.6%]. Age, computer literacy, computer access at health facilities, attitude towards EHRs, awareness about EHRs, perceived benefit, and perceived technology self-efficacy were significantly associated with the overall health care providers’ readiness for the adoption of EHR using a cut point of *P*-value less than 0.05.

**Conclusion:**

Around half of the respondents had a good level of overall healthcare providers’ readiness for the adoption of EHR which was considered inadequate. This finding implied that a huge effort is required to improve readiness before the actual implementation of EHRs. The finding implied that younger-aged groups were more ready for such technology which in turn implied; the older one needs more concern. Enhancing computer literacy, confidence building to raise self-efficacy of such technology, addressing the issue of computer availability at health facilities, building a positive attitude, awareness campaign of EHR, and recognizing the usefulness of such systems were the necessary measures to improve EHR readiness in this setting.

Additionally, further studies are recommended to encompass all types of EHR readiness such as organizational readiness, technology readiness, societal readiness, and so on. Additionally, exploring the healthcare provider opinion with qualitative study and extending the proposed study to other implementation settings are recommended to be addressed by future works.

**Supplementary Information:**

The online version contains supplementary material available at 10.1186/s12913-022-07688-x.

## Introduction

In this digital era, information communication technologies (ICTs) have been widely used and expanded in the healthcare industry to manage big data and voluminous health information [1]. Health information technology (HIT) is a broad concept that encompasses the use of ICT for health care services delivery [2]. The interest in implementing HIT in the healthcare system is growing rapidly worldwide during the past ten years [3, 4]. Among the different HIT initiatives in the international healthcare system, the EHR is seen as the backbone that supports the integration of various e-health tools [5].

According to the International Organization for Standardization (ISO), EHR means a repository of patient data in digital form, stored and exchanged securely, and accessible by multiple authorized users [6]. Adoption of those systems in the healthcare organization has several benefits including minimizing cost, increasing revenue, improving patient care, reducing the need for file space, data confidentiality, reducing medical errors, central patient data management, and sharing medical information [7–12].

Many countries in the world tried to implement an EHR which lead to a 46% global increase in the past five years [13–18]. However, more than 50% of those electronic record projects in the world were failed without providing their targeted benefits [19]. Particularly, the implementation of EHR remains a major challenge in the healthcare systems of low-income countries [20–23]. Only 15% of low-income countries have nationally adopted electronic record systems in health institutions [24]. Sub-Saharan countries are relatively more likely lagging in adopting those technologies due to the digital divide and different social issues such as electrical power interruption, health professionals’ technology resistance, and ICT infrastructure [18, 25–29].

On the other hand, literature shows tremendous progress in using and deploying EHR in a few countries of this region [30, 31]. Previous experience in the implementation of digital technologies implied EHR is feasible and cost-effective in resource-limited settings [32–37].

A readiness assessment is a prominent solution for the effective implementation of EHRs which portrays a proper image of existing conditions and the preparedness of health institutions and health professionals for the new system [38, 39]. Previous frameworks were conceptualized healthcare providers’ readiness as among different types of e-health readiness which is an integrated of core readiness and engagement readiness [33, 40–43].

Studies were conducted to assess healthcare providers’ readiness to adopt EHR in developed countries [44–47]. However, we argue that those results are not representative of the status in low-income countries’ settings due to the variation in digital technology penetration. On the other hand, studies were conducted to assess the organizational readiness level of EHR in low-income countries settings which were rated “inadequate readiness level” [41, 44, 48–51]. But, pre-implementation assessment in this setting rarely addressed EHR readiness in healthcare providers’ context, which was blamed among the main reason for the failure of those EHR projects [52–54].

In Ethiopia, studies had been carried out on knowledge, attitude, willingness, and acceptance of electronic records [18, 25, 55–62]. To the best of the researcher’s knowledge, very few studies were conducted on the readiness of electronic medical records (EMRs) at purposively selected primary health facilities in northwest Ethiopia which showed the level of readiness was 54.1% [63] and 62.3% [64]. Nevertheless, there were limited reports specifically on country-wide healthcare providers’ EHR readiness. Different efforts have been made to implement a digital health information system (HIS) in Ethiopia. The government of Ethiopia deployed District Health Information System 2 (DHIS2) to manage the national reporting system only. Additionally, the ministry of health (MOH) deployed EMR in a few Hospitals of central, eastern, and northern parts of Ethiopia as a pilot. But, the plans to scale up those EMRs to other hospitals were failed [65].

Moreover, the government of Ethiopia proposed to implement country-wide EHR which initiated the authors of this research to investigate the readiness level before the actual implementation. Previous progress in digitalization of healthcare such as the implementation of DHIS2, electronic community health information system (e-CHIS), and EMR in a few hospitals indicated that the implementation capability of the country-wide EHR if the pre-implementation activities are handled effectively and supported with research findings [56, 65–67].

The main hindering factors that influence the readiness of healthcare professionals in the implementation of an EHR are: sex [50, 68], age [50, 69–71], knowledge [30, 45, 50, 72–75], attitude [74, 76, 77], awareness [78, 79], innovativeness [75], training [63, 64, 80], computer literacy [50, 63, 72, 74], workload [81], management support [30, 82], experience [40, 44, 46, 57, 63, 64, 72, 83–86], self-efficacy [41, 68, 87], perceived benefit [30, 77, 88, 89], computer use, and internet access [90].

The authors of this study believed that investigating the user’s readiness and the necessary measurements to be taken is crucial for effective interventions before the adoption of such systems in Ethiopia. This study also enabled policy-makers in resource-limited settings to understand users’ needs before to having the actual system implementation. Therefore, this study was designed first; to show the level of EHR readiness, second, to assess factors impeding health care providers’ readiness towards EHR adoption in southwest Ethiopia.

## Methods

### Study design and setting

The institutional-based cross-sectional study design was conducted from September 1 to October 30, 2020. The study was conducted at public hospitals in Illu Aba Bora and Buno Bedele Zones, Oromia Region, Southwest Ethiopia. The capital city of Illu Aba Bora and Buno Bedele is Mettu and Bedele respectively. Mettu and Bedele cities are located 600 km and 580 km away from Addis Ababa, the capital city of Ethiopia. The two zones were demarcated as one administrative zone until recent times. The total population of those zones was 1,271,609. Among this, 636,986 and 634,623 were males and females respectively. Farming is the predominant source of income in the community to lead their life. In terms of infrastructure development, there were 5 hospitals (1 referral hospital, 1 General hospital, and 3 Primary hospitals) within the two zones. A total of 41 and 23 health centers were found in Illua Aba Bora and Buno Bedelle Zones respectively.

### Study participants, sample size, and sampling procedures

All health professionals permanently working in Illu Aba Bora and Buno Bedele zones, southwest Ethiopia were eligible in this study. The sample size was calculated assuming the prevalence of healthcare providers’ EHR readiness level to be 50% since the study wasn’t found specifically on EHR readiness similar to the current study setting. We also consider the following assumptions: a 95% level of confidence, a 5% of margin of error, and a 5% of non-response rate. Finally, a sample size of 423 was obtained. Five fully functional hospitals and 1,398 healthcare providers working in those hospitals were found in Illu Aba Bora and Buno Bedele Zones during the data collection period of the study. We were proportionally allocated the total sample size, 423, to those five public hospitals found in the two zones. Then, health professionals were randomly selected in those hospitals (See Supplementary file 1 for detail).

### Data collection tools and procedure

Data were collected using a pretested self-administered questionnaire. The questionnaire was adapted from related e-health studies conducted elsewhere in the world [30, 49, 50, 63, 85]. Pretested self-administered questioners were used. The content of the questioners contained five parts. Part1 assessed socio-demographic factors, part two was about behavioral and technical factors, part three assessed technological factors, and part four was about organizational factors. The last section of the questioners was about EHR readiness (13 item questions). A total of 76 item questioners, which took from 10–30 min to fill were used.

The validity of the questioners was checked using expert validity. The reliability was also checked using Cronbach alpha’s coefficient (e.g. overall Cronbach alpha for healthcare providers readiness = 0.86). The investigators provided two-day training for data collectors and supervisors. Two master holder health professionals as supervisors and 10 HITs/statisticians as data collectors participated in data the collection process. During data collection, participants were informed about the objective and processes of the study and the confidentiality of the information.

### Measurements

In this study, we used the mean and median scores to dichotomize our variables such as EHR readiness, knowledge, attitude, awareness, computer literacy, personal innovativeness in information technology(PIIT), self-efficacy, perceived benefit, and management support. If the variable was normally distributed, we computed the mean score. On the other hand, we used the median score if the variable was not normally distributed [50, 64, 91].

#### Healthcare providers

All professionals working at health facilities and who have at least a diploma certificate in any health and medicine fields were operationalized as healthcare providers in this study [11, 63].

#### EHR readiness

The preparedness of healthcare providers to embrace changes brought by the introduction of a computerized system. In this study, EHR readiness was defined as stakeholders’ readiness about EHRs [33]. Healthcare providers incorporate both engagement and core readiness. Accordingly, we were concerned about healthcare providers’ overall readiness. So, we used a sum-up of engagement and core readiness [33, 50].

A total of 13 item Likert scale questioners were used in which 8 of those about core readiness and 5 of the items about engagement readiness. Engagement Readiness refers to the involvement of healthcare providers using EHR [33, 41]. Core Readiness refers to the need of EHR related to current conditions which include the importance of needs, planning, and accessibility such as appropriateness of EHR technology and integration of this technology with existing healthcare services, as well related to the core attributes of the target population that leads to the need for change [33, 41, 50]. In this case, respondents who scored the mean and above were considered as having a good level of healthcare providers’ readiness to adopt EHR. On the other hand, respondents who were scored below the mean were considered as having a poor level of healthcare providers’ readiness to adopt EHR [63, 64].

#### EHR knowledge

We used fourteen Likert scale questions that deal with the three aspects of EHR including what is EHR, fields of its application, and methods of its use. The questions ranged from “1=strongly disagree to 5= strongly agree”. Respondents were responded by “yes”, “No” or “Do not know”. Knowledge score was calculated as follows: 1 point for a correct answer and 0 points for don’t know & incorrect answer. Respondents who scored the mean and above were considered as having good knowledge, whereas those who scored below the mean were considered as having poor knowledge [45, 50, 78].

#### EHR attitude

Professional feeling towards using EHR. The attitude of the study participants was assessed by using six-item questions rated on a five-point Likert scale that ranged from "1 = strongly disagree" to "5 = strongly agree". Then, the scores of the Likert scale statement were dichotomized into two. Study participants who scored equal to and above the mean were considered as having a favorable attitude whereas participants who scored below the mean were considered as having an unfavorable attitude [45, 76, 92, 93].

#### Awareness toward EHR

This was measured by three Likert scale questions. Respondents were asked if they were aware of the relevant application of computerization in health care, the existence of EHR systems, and the benefit of EHR technologies. Study participants who scored the median and above were considered as aware of EHRs [74, 79].

#### Computer literacy

It was defined as the computer-related knowledge in a capacity to obtain, communicate, process, and understand the basic information to make appropriate health decisions. It was measured by five items of Likert scale questions ranging from”1 = strongly disagree to 5 = strongly agree”. Respondents who scored median and above were considered as having high computer literacy and those who were scored below the median score were indicated as having low computer literacy [63, 64].

#### Perceived self-efficacy

We used a modified computer self-efficacy scale which was adapted to be used by clinicians. This is a perceived technology self-efficacy to adopt electronic health records. Participants were asked to rate their confidence in using new EHR technologies, if available. A total of ten Likert scale questions ranging from “1 = strongly disagree to 5 = strongly agree” were used. Respondents who scored mean and above were considered as having good efficacy [41, 87, 94].

#### PIIT

It indicated "the willingness of an individual to try out any new information technology (IT)”. It was measured by four-item Likert scale questions ranging from “1 = strongly disagree to 5 = strongly agree”. Respondents who scored mean and above were considered innovative in IT [70].

#### Perceived benefit

This is assessing the participants’ subjective expected benefit from adopting the EHR system. It was measured by fourteen Likert scale questions ranging from “1 = no value to 5 = very important value” [41]. Respondents who scored mean and above were recoded as valuable and those who scored below mean were recoded as not valuable [41, 49].

#### Top management support

The necessary supports provided by senior managers that were measured with four-item Likert scale questions ranged from “1 = strongly disagree to 5 = strongly agree”. Respondents who scored mean and above were considered as having good management support and those who scored below the mean were considered as having poor management support [49].

### Data processing and analysis

Data were entered checked, cleaned, and edited using Epi-data version 4.6. Then, the data were exported to STATA version 14.1 for further analysis. Binary logistic regression analysis was conducted to discover the effect of each study variable on the outcome variable. Predictor variables having a *P*-value < 0.2 on the bivariate analysis were entered into a multivariable logistic regression analysis to check for confounding effects. A forward stepwise technique was applied to identify explanatory variables that have a significant association with the outcome variables to build the multilevel Model. The strength of association was described at 95% CI and *P*-value less than 0.05 was considered as a cut point for multivariable logistic regression analysis. A multicollinearity test was conducted for the model and none of the variables scored above 10 for the test statistic.

## Results

### Socio-demographic characteristics

From 423 distributed questioners, 386 responses were received (with a response rate of 91.3%). Two hundred twenty-five (58.3%) of the respondents were males. In terms of health facility, 153(39.6%), 101(26.2%), and 132(34.2%) of the respondents were working at primary hospitals, general hospitals, and referral hospitals respectively. The mean age of the participants was 29.53 ± 8.7 years. The majority, 263(68.1%) of the respondents were first-degree holders.

This study revealed that the majority, 110(28.5%) of the respondents were nurses. In terms of the working unit, 149(38.6%) and 125(32.4%) of the respondents were working in IPD and OPD wards respectively. One hundred sixty-three (42.2%) of the respondents had less than six-year of experience and the mean of experience was 6.8 ± 5.3 years. On the other hand, 145(37.6%) of the respondents were responded as they had a workload in their facilities. Among the total respondents, 84(21.8%) were management members. In terms of their monthly income, 260(67.4%) of the study participants had salaries greater than 5,000 ETB (See Table 1 for details).

**Table 1:** Socio-demographic characteristics of healthcare providers working at public hospitals in Southwest Ethiopia, 2021

Variable	Category	Frequency (#)		Percent (%)
Sex	Female		161	41.7%
	Male		225	58.3%
Type of health facility	Primary hospital		153	39.6%
	General hospital		101	26.2%
	Referral hospital		132	34.2%
Age	21–30		214	55.4%
	31–50		135	35.0%
	> = 51		37	9.6%
Religion	Orthodox		122	31.6%
	Muslim		72	18.6%
	Protestant		179	46.4%
	Others		13	3.4%
Educational level	Diploma		99	25.7%
	B.Sc. degree		263	68.1%
	Master and above		24	6.2%
Profession/educational background	Medicine		51	13.2%
	Nurse		110	28.5%
	Midwife		89	23.1%
	Public health officer		45	11.6%
	Pharmacy		37	9.6%
	Laboratory		32	8.3%
	Others		22	5.7%
Ward	OPD		125	32.4%
	IPD		149	38.6%
	MCH		67	17.3%
	Others		45	11.7%
Work experience	Less than 6		163	42.2%
	6–10		137	35.5%
	Greater than 11		86	22.3%
Workload	No		241	62.4%
	Yes		145	37.6%
Management(mgt) member	Staff		302	78.2%
	Mgt Member		84	21.8%
Salary(in ETB)	< = 5,000 ETB		126	32.6%
	> 5,000 ETB		260	67.4%

### Behavioral and technical factors

In this study, behavioral and technical factors were incorporated awareness, EHR knowledge, Attitude toward EHR, PIIT, computer skill, perceived benefit, and perceived self-efficacy. Accordingly, 335(86.8%) of the respondents had awareness about EHR. On the other hand, 161(41.7%) and 181 (46.9%) of the respondents had good knowledge and favorable attitude respectively. The PIIT was 145(37.6%). Two hundred four (52.8%) of the respondents had sufficient computer Literacy. About, 183(47.4%) of the respondents had high perceived self-efficacy (See Table 2 for details).

**Table 2:** Behavioral and technical factors among healthcare providers working at public hospitals in Southwest Ethiopia, 2021

Variable	Category	Frequency (#)	Percent (%)
Awareness	Not aware	51	13.2%
	Aware	335	86.8%
EHR knowledge	Poor	225	58.3%
	Good	161	41.7%
Attitude toward EHR	Unfavorable	205	53.1%
	Favorable	181	46.9%
PIIT	Poor	241	62.4%
	Good	145	37.6%
Computer literacy	insufficient	182	47.2%
	sufficient	204	52.8%
Perceived benefit	No	29	7.5%
	Yes	357	92.5%
self-efficacy	Low	203	52.6%
	High	183	47.4%

### Organizational and access to basic technology-related factors

Among the total respondents, only 91(23.6%) of them agreed that they had IT technical support in their health facility. In terms of superior management support, 148(38.3%) of the respondents got this support. The result of this study implied that almost no EHR training, which was 23(6.0%). Functional computer access in the working unit was 107(27.7%). On the other hand, only 58(15.0%) had an EHR manual in the working unit. One hundred thirty-four (34.7%) of the respondents accessed the internet service.

Additionally, 284(26.4%) of the respondents confirmed that they had access to uninterrupted electric power and 83(21.5%) of those respondents used software applications in the department. The report also implied only 34(8.8%) of the respondents used a computer at work daily. Two hundred twenty-nine (59.3%) of the respondents had experience in using email for exchanging information (See Table 3 for details).

**Table 3:** Organizational and access to basic technology related factors among health professionals in Southwestern Ethiopia, 2021

Variable	Category	Frequency (#)	Percent (%)
IT technical support	No	295	76.4%
	Yes	91	23.6%
Superior management support	No	238	61.7%
	Yes	148	38.3%
EHR Training	No	363	94.0%
	Yes	23	6.0%
Availability of functional computer at working unit	No	279	72.3%
	Yes	107	27.7%
EHR manual in the working unit	No	328	85.0%
	Yes	58	15.0%
Internet access in the working unit	No	252	65.3%
	Yes	134	34.7%
Uninterrupted electric power	No	284	73.6%
	Yes	102	26.4%
Software application in the department	No	303	78.5%
	Yes	83	21.5%
How often do you use a computer at work?	Never	98	25.4
	Sometimes	254	65.8
	Daily	34	8.8
Experience in using email for information exchange	No	157	40.7%
	Yes	229	59.3%

### Readiness to adopt electronic health record

**Fig. 1:**
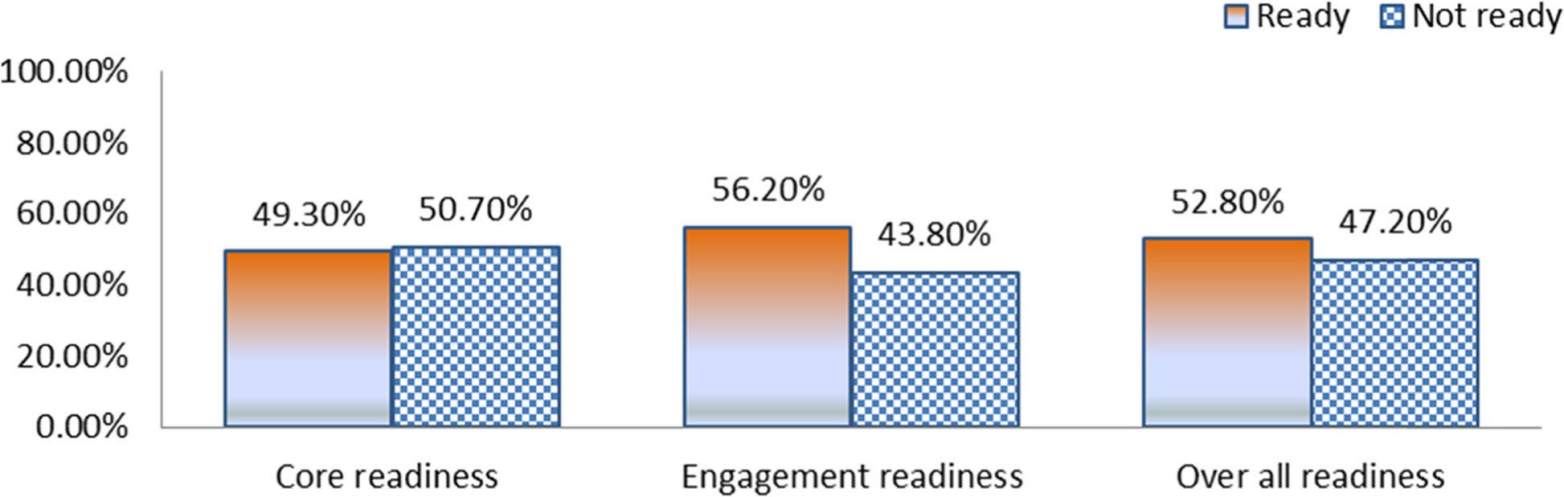
Readiness to adopt electronic health record

In this study, the overall EHRs adoption readiness was 52.8% (*n* = 204) [95% CI of 47.9% to 56.6%]. Among those, 190 (49.3%) had core readiness whereas 217 (56.2%) had engagement readiness (See Fig. 1 for detail).

### Factors associated with readiness to adopt EHR

From the total variables entered into the bi-variable logistic regression model, age, computer skill, computer access, attitude, knowledge, awareness, perceived benefit, perceived self-efficacy, PIIT, training, and availability of technical support were factors associated with readiness to adopt EHR in the bi-variable analysis at *P*-value less than 0.02. Consequently, those variables were subjected to the multivariable logistic regression analysis to control potential confounders.

In the multivariate logistic regression analysis, respondents who were bellow age groups of less than 30 [AOR = 2.25, 95% CI (1.33–3.82)], computer literacy [AOR = 5.02, 95% CI (2.90–8.71)], computer access at health facilities[AOR = 2.76, 95% CI(1.44–5.27)], attitude towards EHRs [AOR = 4.60, 95% CI(2.63–8.04)], awareness about EHRs [AOR = 1.79, 95% CI(1.93–4.18)], perceived benefit about EHRs [AOR = 4.59, 95% CI(1.62–12.99)], and perceived technology self-efficacy [AOR = 4.7, 95% CI(2.71–8.17)] were significantly associated with overall health care providers readiness for the adoption of EHR at *P*-value less than 0.05 (See Table 4 for detail).

**Table 4:** Multivariable logistic regression factors associated with the healthcare providers’ readiness level

Variables	Category	EHR readiness level		Odds Ratio (95% CI)	
		**Poor**	**Good**	**Crude (COR)**	**Adjusted (AOR)**
Age	Above 30	97	75	1	1
	Bellow 30	85	129	1.96(1.31,2.95)	2.25(1.33,3.82)*
Computer literacy	Poor	131	51	1	1
	Good	53	151	7.32(4.67,11.48)	5.02(2.90, 8.71)*
Computer access	No	160	119	1	1
	Yes	22	85	5.19(3.07,8.79)	2.76(1.44,5.27) *
Attitude toward EHR	Unfavorable	125	80	1	1
	Favorable	57	124	3.40(2.23,5.18)	4.60(2.63,8.04)*
EHR knowledge	Poor knowledge	127	98	1	1
	Good knowledge	55	106	2.49(1.64,3.79)	1.20(0.71,2.05)
Awareness towards EHR	Not aware	31	20	1	
	Aware	151	184	1.90(1.03,3.45)	1.79(1.93,4.18)*
Perceived Innovativeness	No	131	110	1	1
	Yes	51	94	2.19(1.43,3.36)	0.76(0.42,1.40)
Perceived Benefit	Not beneficial	24	5	1	1
	Beneficial	180	177	4.72(1.76,12.65)	4.59(1.62,12.99)*
Perceived self-efficacy	Low	130	73	1	1
	High	52	131	4.49(2.92,6.90)	4.7(2.71,8.17)*
EHR training	No	176	187	1	1
	Yes	6	17	2.67(1.03,6.92)	1.92(0.61,6.01)
Technical support	No	151	144	1	1
	Yes	31	60	2.03(1.24, 3.31)	1.87(0.95, 3.68)

^*^P-value < 0.05 for multivariable analysis, 1 = reference category

## Discussion

This research assessed healthcare providers’ EHR readiness and associated factors in Southwest Ethiopia. Even if there are different types of EHR readiness, we, the authors of this research, focused on healthcare providers’ readiness. The overall healthcare providers’ readiness consisted of engagement readiness and core readiness. The overall good readiness level to implement EHR was 52.8% (*n* = 204) [95% CI of 47.9% to 56.6%]. One hundred ninety (49.3%) had a good level of core readiness whereas 217 (56.2%) had a good level of engagement readiness. Our finding corresponds with previous studies in Northern Ethiopia in which e-health readiness specifically to EMR was 54.1% [63], and also another study in Northern Ghana in which the overall readiness to implement EHR was 54.9% [50]. It was also in line with the study conducted in Iran and Myanmar in which the overall EHR readiness level was 56.0% [47] and 54.2% [73] respectively.

Our finding was comparably lower than previous findings of Northern Ethiopia in which the overall readiness of health professionals for an EMR system was 62.3% [64]. This could be due to the study in Northern Ethiopia being conducted at primary health facilities only whereas our study was incorporated primary hospitals, general hospitals, and referral hospitals. Additionally, the study conducted in Northern Ethiopia was using purposively selected primary health facilities which might inflate the report.

The finding was lower than the previous study in California whereas 73.0% [95] of fourth-year medical students were ready in using EHR. This could be due to the study setting and the variation in ICT infrastructure between the two countries. The other possible justification could be in the study of California, the EHR was already deployed but it was in the pre-implementation phase in the current study setting.

The current finding was slightly lower than the study conducted in Iran in which the level of EHR readiness was 57.2% [96]. This variation could be due to the study setting and study participants in which the study in Iran was conducted among nurses only. The other possible justification could be the tool variation and differences in ICT infrastructure. The study in Iran was conducted at one referral hospital only which might be the reason for this discrepancy. On the contrary, the finding in the current study was higher than the other study reported in Iran in which 28.6% [45] were ready for pre-implementation. This variation could be due to the variation in tools used, the study period, and the sampling technique. The study conducted in Iran used a survey of health workers found in one general hospital which might be the other possible reason for this variation.

Readiness to adopt EHRs was interlinked with socio-demographic, behavioral, technical, technological, and organizational factors. Healthcare providers aged below 30 were more likely to be ready to adopt EHR compared to who were aged above 30. This finding was supported by previous reports which stated younger aged healthcare providers were more engaged in using technology [50, 69]. The possible justification for this finding could be the innovative potential of the younger people. This implied that younger people are early adopters of e-health which strengthens previous research findings in e-health and IT technology adoption [71, 97].

The odd of healthcare providers who had favorable attitude were more likely to be ready than those who had unfavorable attitude. The finding was in line with previous studies [45, 74, 76, 77]. The possible justification for this could be the positive evaluation of healthcare providers to those technologies is a driving force to be more eager and committed to engaging in EHR.

Our finding implied healthcare providers who had good awareness were more likely to be ready. This finding was consistent with previous research findings [78, 79]. This implied that awareness campaigns enabled individuals to understand the values of EHRs and to take action.

The study implied that computer literacy played an important role in determining healthcare providers’ readiness to adopt EHRs in which respondents with high computer literacy were more ready than counterparts. This finding was supported by previous reports elsewhere in the world [50, 63, 72, 74]. This could be due to such skills helping to perform tasks in any computerized technology. This implied that computer skill is essential to processing and presenting information in computerized technology like EHR.

The study revealed healthcare providers were more likely to be ready when they believed that EHR is more beneficial. This finding was corresponding with previous research findings [30, 77]. This could be since healthcare providers who considered EHRs as beneficial were more likely to be motivated and be ready.

This study found that healthcare providers who had a good perceived self-efficacy were more likely to be ready. This finding was in line with previous studies elsewhere in the world [41, 68, 87]. This could be due to computer self-efficacy influencing individuals to be confident in their skills and abilities to perform tasks related to computer technology.

Healthcare providers who got computer access at facilities were ready to EHRs. This finding was supported by previous reports elsewhere in the world [63, 64]. This could be due to the availability of computer-enabled ones to practice digital tools. Moreover, computer accesses allow the daily practice of e-health technologies, so it enhances the skill and confidence to engage in EHR.

### Strengths and limitations of the study

This study was the first study in Ethiopia to assess the detailed measurements to be taken in improving healthcare providers’ readiness level before EHR implementation. It also presented some highlights for measurements to be taken before EHR implementation in low-income country settings. However, causality cannot be inferred since the study was cross-sectional. The major limitation of the study was that it didn’t triangulate with qualitative findings. Additionally, it didn’t incorporate other types of readiness for instance organizational and technology readiness since there isn’t a compressive tool to incorporate all types of EHR health readiness.

## Conclusions

Around half of the respondents had a good level of overall readiness for the adoption of EHR which was considered inadequate. This finding implied that a huge effort is required to improve readiness before the actual implementation of EHRs. Particularly above half of the healthcare providers had poor core readiness levels. What we have learned from this statement is that most of the healthcare providers were highly dissatisfied with the existing paper-based system and eager to implement EHR. The finding implied that younger-aged groups were more eager for such technology which in turn implied the older ones need more concern.

Enhancing computer literacy, building their confidence to rise self-efficacy such technology, building a positive attitude, awareness campaign of HER, and recognizing the usefulness of such systems were the necessary measures to improve EHR readiness in this setting. Further studies are recommended to encompass all types of EHR readiness such as organizational readiness, technology readiness, societal readiness, and so on. Additionally, exploring healthcare providers’ opinions with qualitative study and extending the proposed study to other implementation settings are recommended to be addressed by future works.

## Supplementary Information

**Additional file 1. **Sampling procedure.

## Data Availability

The data will be available upon request from the corresponding author.

## Abbreviations

AOR: Adjusted Odds Ratio
CI: Confidence Intervals
COR: Crude Odds Ratio
DHIS2: District Health Information System Two
e-CHIS: Electronic Community Health Information System
e-Health: Electronic Health
EHR: Electronic Health Record System
EMR: Electronic Medical Record
HIS: Health Information System
HIT: Health Information Technology
ICT: Information Communication Technology
ISO: International Organization for Standardization
IT: Information Technology
MOH: Ministry of Health
PIIT: Personal Innovativeness in Information Technology
