# Supplementary material for: Healthcare providers’ readiness for electronic health record adoption: a cross-sectional study during pre-implementation phase
Source: BMC Health Serv Res. 2022 Mar 2;22:282. doi: 10.1186/s12913-022-07688-x (PMC8889777; doi:10.1186/s12913-022-07688-x)
Supplement: Supplementary file 1 — Additional file 1. Sampling procedure. [file 12913_2022_7688_MOESM1_ESM.pdf]

Sampling Procedure and Sample Size

All five (5) hospitals located within the Illu Aba Bora and Buno Bedele zones were approached and used for this study. All healthcare providers permanently working in those five hospitals were included in the study. Sample size was calculated using the population proportion allocation formula, by considering EHR readiness level to be 50% since study wasn't found specifically on EHR readiness in the current study setting. We also considered the following assumptions: a 95% level of confidence, a 5% of margin of error, and a 5% of non-response rate. Finally, a sample size of 423 was obtained. The total sample size was proportionally allocated to each hospital. Study participants were selected using simple random sampling method from all five hospitals. Steps:

1. The total sample size, 423, were allocated proportionally to all five hospitals found in our study setting
2. Healthcare providers were randomly selected from those hospitals until we get the saturation based on the proportional allocated

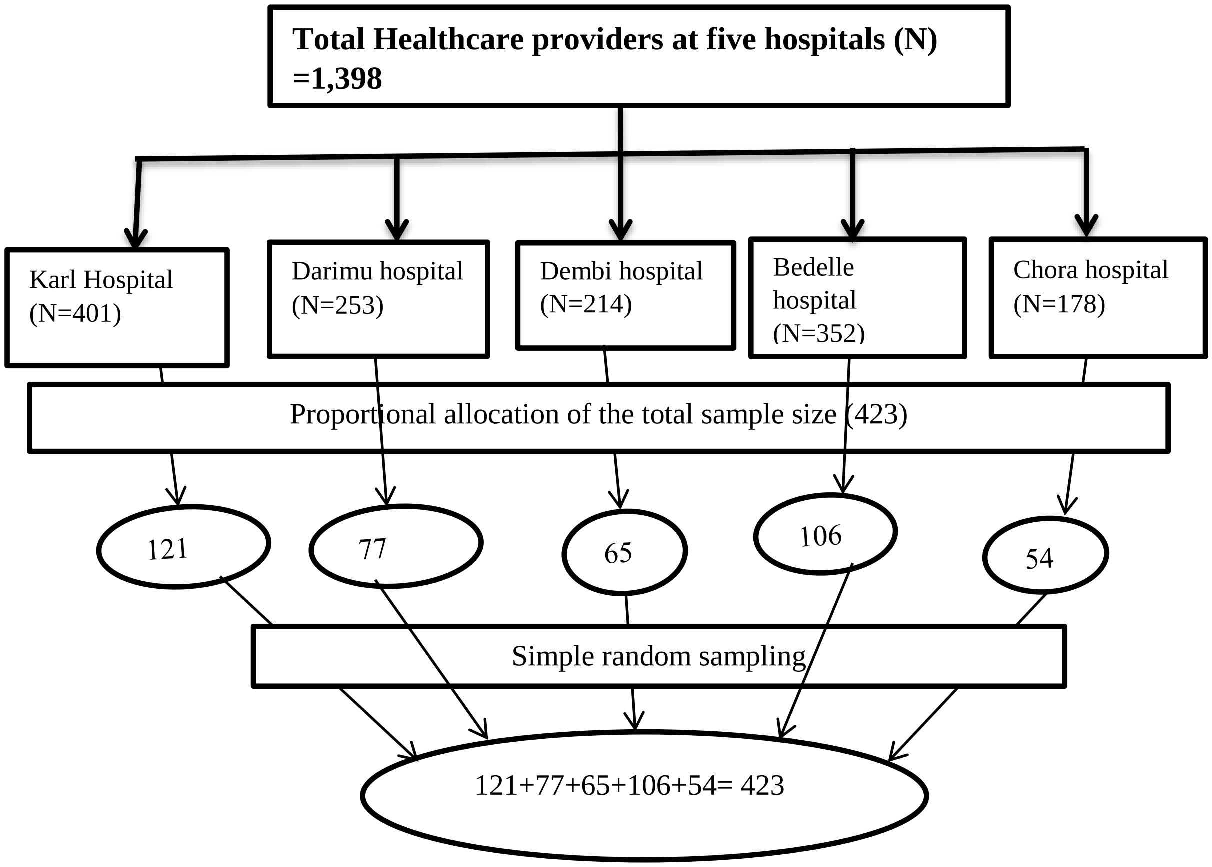

Figure: Sampling procedure of Healthcare providers' readiness to adopt EHR: During Pre-implementation phase in southwestern Ethiopia;
